# Synthetic Soil Aggregates: Bioprinted Habitats for High-Throughput Microbial Metaphenomics

**DOI:** 10.3390/microorganisms10050944

**Published:** 2022-04-30

**Authors:** Darian Smercina, Neerja Zambare, Kirsten Hofmockel, Natalie Sadler, Erin L. Bredeweg, Carrie Nicora, Lye Meng Markillie, Jayde Aufrecht

**Affiliations:** Earth and Biological Sciences Directorate, Pacific Northwest National Laboratory, Richland, WA 99352, USA; neerja.zambare@pnnl.gov (N.Z.); kirsten.hofmockel@pnnl.gov (K.H.); natalie.sadler@pnnl.gov (N.S.); erin.bredeweg@pnnl.gov (E.L.B.); carrie.nicora@pnnl.gov (C.N.); meng.markillie@pnnl.gov (L.M.M.)

**Keywords:** soil, microbiome, bioprinting, multi-omics, metaphenome, soil aggregates, biogeochemistry, bacteria, fungi, synthetic habitat

## Abstract

The dynamics of microbial processes are difficult to study in natural soil, owing to the small spatial scales on which microorganisms operate and to the opacity and chemical complexity of the soil habitat. To circumvent these challenges, we have created a 3D-bioprinted habitat that mimics aspects of natural soil aggregates while providing a chemically defined and translucent alternative culturing method for soil microorganisms. Our Synthetic Soil Aggregates (SSAs) retain the porosity, permeability, and patchy resource distribution of natural soil aggregates—parameters that are expected to influence emergent microbial community interactions. We demonstrate the printability and viability of several different microorganisms within SSAs and show how the SSAs can be integrated into a multi-omics workflow for single SSA resolution genomics, metabolomics, proteomics, lipidomics, and biogeochemical assays. We study the impact of the structured habitat on the distribution of a model co-culture microbial community and find that it is significantly different from the spatial organization of the same community in liquid culture, indicating a potential for SSAs to reproduce naturally occurring emergent community phenotypes. The SSAs have the potential as a tool to help researchers quantify microbial scale processes in situ and achieve high-resolution data from the interplay between environmental properties and microbial ecology.

## 1. Introduction

Microorganisms in the soil, collectively referred to as the soil microbiome, govern the biogeochemical cycles necessary for all life [1]. By accurately quantifying and modeling soil microbial processes, researchers could improve global carbon projections and better predict the impacts of climate change [2,3]. However, upscaling microbial processes to ecosystem level fluxes remains a scientific challenge due to a poor correlation between microbiome structure and properties (e.g., multi-omics data) and microbial processes (e.g., rates of biogeochemical transformations) [3,4]. To compound the challenge of upscaling microbial processes, there is also an insufficient understanding of how environmental conditions shape the microbiome structure, properties, and processes. This knowledge gap arises, in part, from the lack of experimental data characterizing soils at the sub-millimeter spatial resolution that quantifying microbial processes requires [5]. 

One option for characterizing soils at sub-millimeter scales is to study individual soil aggregates or physically bound soil particles ranging in size from a few hundred µm to a few mm. Soil aggregates are a fundamental component of soil structure and have been described as both evolutionary and biogeochemical incubators of the soil microbiome [6,7]. In contrast to bulk soil analyses, studies focused on individual soil aggregates show that the metagenomic structure of microbial communities within discrete soil aggregates are distinct and that enzyme activity varies with aggregate size [8,9,10]. Thus, soil aggregates represent a key functional unit of soil and a critical area of research for understanding microbial processes [11]. However, the characterization of microbial community dynamics within soil aggregates presents many technical challenges. For example, the opacity of soil aggregates precludes the ability to non-destructively image microbes within the aggregate pore spaces; the variation in physiochemical properties between aggregates can confound data interpretation; and soil sieving methods can bias the enzyme activity within individual aggregates [8,9].

To bypass the challenges of studying microbial dynamics in natural soils, we present an alternative method for characterizing the soil microbiome on a sub-millimeter scale: Synthetic Soil Aggregates (SSAs). SSAs are fabricated using 3D bioprinting to directly embed microbial cells within a translucent and porous hydrogel-based habitat. Three-dimensional bioprinting technology is increasingly used for the study of mammalian cell development; thus, the methods are well established [12,13]. However, to date, this state-of-the-art technique has not been widely applied to the study of microorganisms in other environments such as soil. Here, we demonstrate how SSAs can be rapidly bioprinted for high-throughput characterization of microbial communities residing in the internal SSA pore network. We established a multi-modal workflow for integrating SSAs with multi-omics analyses including genomics, metabolomics, proteomics, and lipidomics. Finally, we demonstrate that SSAs can be used to characterize microbial spatial interactions using a model co-culture of *Escherichia coli* and *Pseudomonas fluorescens*.

## 2. Materials and Methods

### 2.1. Bioprinting a Synthetic Soil Aggregate 

A commercially available extrusion-based bioprinter (Inkredible+, Cellink, Bico Group, Boston, MA, USA) was used to fabricate the SSAs. The hydrogel-based bio-ink used to create the SSAs was a commercial formulation of gelatin methacrylate (45–55% methacrylation) with nanofibrillated cellulose and 0.25% lithium phenyl-2,4,6- trimethylbenzoyl phosphinate photoinitator (GelMA C, Cellink, Bico Group, Boston, MA, USA). The hydrogel was pre-heated to 35 °C in a water bath for approximately 10 min before it was loaded into the pre-heated bioprinter cartridge. To print a single SSA, a custom *gcode* file rendered in HeartWare (Cellink, Bico Group) directed the syringe to the substrate’s surface (i.e., a Petri dish or 96-well plate) and turned the printhead on for 3 s. Using an air pressure of 9 kPa and a 21-gauge blunt needle tip, this design resulted in a convex 3D SSA with a diameter of approximately 2 mm (coefficient of variance: 0.13 for *n* = 10). After printing, the SSAs were exposed to UV light at 365 nm for 30 s to crosslink the gelatin methacrylate. 

### 2.2. Scanning Electron Microscopy

To prepare the SSAs for imaging via a scanning electron microscope (SEM), the SSAs were printed into a small Petri dish and UV-cured as previously described. Immediately after curing, the Petri dish was flash frozen in liquid nitrogen and transferred to a vacuum lyophilizer to dry overnight. Using a razor blade, the lyophilized SSAs were carefully transferred to double-sided tape mounted on a microscope slide and then sputtered with a thin layer of gold (~5 nm) to make them electrically conductive and then imaged using a tabletop SEM (TM1000, Hitachi, Ltd., Tokyo, Japan).

### 2.3. Pore Size Characterization

A representative 8-bit SEM image of an SSA (250× magnification) was analyzed in ImageJ to extract pore size data. The MorpholibJ plugin was used to segment the image based on morphological segmentation with the “border image” parameter selected and a tolerance of 25 (Figure S1) [14,15]. The max Feret diameter of each segmented pore was analyzed and converted from pixels to microns. 

### 2.4. Diffusion of TRTIC-Dextran in GelMA C

GelMA C was extruded by hand from a syringe into an open fluidic channel (50 mm × 2.5 mm × 0.8 mm, Sticky slides, Ibidi). Care was taken to completely fill the width and height of the channel with hydrogel to an approximate 10 mm length of the channel. The hydrogel was then cured for 1 min at 365 nm UV light. The paper seal was then removed from the fluidic device to reveal the adhesive layer, and a glass microscope slide was reversibly attached to the Sticky slide to create a closed fluidic channel. De-ionized water was used to pre-fill the channel, and care was taken to avoid trapping air bubbles. Forty microliters of 4.4 kDa TRITC-dextran (1 mg mL^−1^, Sigma, St. Louis, MO, USA) was loaded into one inlet of the Sticky slide, and fluorescence was imaged every 5 min using a Zeiss Axio Zoom to track the diffusion of the fluorescent molecule through the hydrogel. V16 microscope (Ex: 545 nm; Em: 572 nm). Microscope images (16-bit) were analyzed in ImageJ by generating a greyscale intensity plot profile along a line originating at the front edge of the hydrogel and extending approximately 5 mm into the hydrogel. 

Within each time interval, the fluorescence intensity (i.e., greyscale intensity) along the plot profile was normalized by the fluorescence intensity at the front edge of the hydrogel, producing a value of C_x_/C_1_. This measurement was performed under the assumptions that (1) the TRITC-Dextran was concentrated enough in the channel inlet to remain constant over time and (2) the concentration of TRITC-dextran was homogenous between the device inlet and the leading edge of the hydrogel. To ensure that the second assumption was accurate, we used images from later time points (2 h and 3 h) to calculate diffusion coefficients to provide time for the 4.4 kDa TRITC-dextran to diffuse from the inlet to the edge of the hydrogel. The inverse complementary error function was applied to the C_x_/C_1_ values and the diffusion coefficient (D_coeff__._) at each value of x was solved for according to: $D_{coeff.}=\frac{x^{2}}{4tX^{2}}$ [16]. The average diffusion coefficient was determined for C_x_/C_1_ values larger than 0.25 to exclude noisy background levels in greyscale intensity. 

### 2.5. Microbial Strains and Culture Conditions

Five microorganisms were used to validate the bioprinting capability, including *Escherichia coli Dch1A: mScarlet RFP* and *Pseudomonas fluorescens SBW25*: *GFP* [17], *Escherichia coli* MJK2: GFP [18,19], *Paenibacillus* *polymyxa* DSM 36 [20], and *Neurospora crassa*: tdTomato [21]. These organisms represent a diverse array of microorganisms including Gram-negative bacteria, Gram-positive bacteria, and fungi, and all, except *P. polymyxa*, express constitutive production of a fluorescent protein. Culture conditions differed depending on needs of each microorganism. *E. coli* RFP, *P. fluorescens*, and *P. polymyxa* were cultured from frozen stock in Luria Broth (LB) at 25˚C overnight. *E. coli* GFP was cultured from frozen stock in LB broth supplemented with 10 µg mL^−1^ gentamicin, 50 mM L-arabinose, and 10 μM nickel chloride at 25°C overnight. For the ureolysis biosensor experiment (detailed below), growth media for *E. coli* GFP were supplemented with 10 g L^−1^ urea and Bromothymol blue was used to observe the pH change. *N. crassa* conidia were generated by inoculating a 125 mL Erlenmeyer flask containing 20 mL Vogel’s agar with 2% glucose for 7 days, followed by collection with water, and removal of hyphae by gentle swinging-bucket centrifugation in a table-top centrifuge. For experiments, collected conidia were cultured in Vogel’s medium with 1% glucose at 25 °C for 12 h.

### 2.6. Validation of Microbial Viability within Synthetic Soil Aggregates

To test microbial printability, strains within their appropriate media were diluted to an optical density at 600 nm of 0.1 and then mixed in a 1:10 ratio with preheated GelMA C using sterile luer-lock syringes and working within a bio-safety cabinet. To maintain sterility during the bio-printing process, sterile needle tips, cartridges, and substrates (e.g., Petri dish or 96-well plate) were used in conjunction with the HEPA 13 air filter built into the bioprinter housing. Inoculated GelMA C was then printed as SSAs using the soil aggregate. G-code design into 60 mm diameter Petri dishes. Printed SSAs were UV-cured (365 nm) for 30 s and then surrounded with ~3 mL of species-appropriate media to prevent drying and provide adequate resource access. Petri dishes were then sealed with parafilm and incubated at 25 °C for up to 72 h. Metrics of successful printing compatibility were based on demonstrated growth of each species. *E. coli* RFP growth was confirmed in two ways. First, growth curves based on optical density at 600 nm (OD600) were measured using a 48 h kinetic read with 10 min intervals on a Synergy H1 plate reader (BioTek Instruments, Inc., Winooski, VT, USA). For these tests, single SSAs were printed on individual wells of a 96-well plate and then surrounded with 200 µL of LB media. Second, growth was visually confirmed on a Zeiss LSM 710 confocal fluorescent microscope (RFP channel—laser: 561 nm) using end point images taken at 0, 24, and 48 h after printing. A relative increase in fluorescent bacteria was considered a positive response and an indication of bioprinting compatibility. Similarly, *P. fluorescens, N. crassa, P. polymyxa,* and *E. coli* GFP successful bioprinting was validated using end-point fluorescent microscope images taken at 0, 24, 48, and 72 h after printing as appropriate for growth. *P. fluorescens* and *E. coli* GFP were imaged using the GFP channel (laser: 488 nm), and *N. crassa* was imaged using tdTomato settings (laser: 550 nm). *P. polymyxa*, a non-fluorescent strain, was visualized using *Bac*Light live/dead stain (Invitrogen, Waltham, MA, USA) and imaged on two channels: live–green (laser: 488 nm) and dead-red (laser: 595 nm). 

### 2.7. Multi-Omic Analysis of Synthetic Soil Aggregates

SSAs containing pure cultures or co-cultures of *E. coli* RFP and *P. fluorescens* were printed into Petri dishes, and then ~3 mL of MOPS minimal media was pipetted into the Petri dish to surround the SSAs [17]. Cells were incubated overnight, as described above, and then pelleted and resuspended in MOPS media at an optical density of 1.0 measured at 600 nm on a Nanodrop. The bacterial cells from each species were mixed with the GelMA C hydrogel at a ratio of 1:200 (volume of culture to volume of hydrogel). For co-culture SSAs, bacterial cells for each species were mixed with GelMA C separately in sequence such that cells from both species were contained within the same hydrogel. Two nutrient sources were tested by either mixing insoluble 0.2% w/v chitin into the hydrogel or by adding soluble 0.2% N-acetyl-β-D-glucosamine (NAG) to the surrounding MOPS media. SSAs were incubated for 24 or 48 h after printing, as described below, then flash-frozen on liquid nitrogen and stored at −80 °C until multi-omics analysis, including genomic, metabolomic, lipidomic, and proteomic analyses. 

The potential for microbial DNA extraction from SSAs was tested using two commercially available DNA extraction kits including Qiagen DNeasy PowerSoil kit (QIAGEN, Germantown, MD, USA) and Qiagen DNeasy Powerlyzer Microbial kit (QIAGEN, Germantown, MD, USA). The kit’s recommended procedures were followed with some minor adaptations, including an increase in the bead beating step from 10 min (kit recommended) to 15 min for both kits to ensure full disruption of the SSA and a decrease in final elution volume from 100 µL to 50 µL for the PowerSoil kit. *E. coli* RFP DNA was extracted from single SSAs or two SSAs pooled in a single extraction. Growth periods were either 24 or 48 h for single SSAs and 48 h for pooled SSAs. Two replicates were collected from each growth period, for single or pooled SSAs, and for each DNA kit. In total, DNA was extracted from 8 samples. DNA quality (260/280 nm ratio) and concentration (ng µL^−1^) were measured using a Take3 micro-volume plate (BioTek Instruments, Inc., Winooski, VT, USA) and read on a Synergy H1 plate reader.

To confirm that extracted DNA was from bacterial cultures and not contamination, 16S rRNA was amplified using the Ion 16S Metagenomics kit (Life Technologies, Carlsbad, CA, USA) following the recommended protocol. This kit provides two sets of primers: set 1 targets the V2-4-8 region, and set 2 targets the V3-6, 7-9. Each primer set was tested on four samples. Set 1 primers were tested on single SSAs extracted using both DNA kits, while set 2 was tested on single and pooled SSAs extracted using the Powersoil kit. Amplification was confirmed on an Agilent 2100 bioanalyzer (Agilent, Santa Clara, CA, USA) where a positive result was observed at ~300 bp for primer set 1 and ~200 bp bands for primer set 2. In addition to 16S amplification, unamplified DNA fragment size was measured on an Agilent 5200 fragment analyzer (Agilent, Santa Clara, CA, USA).

MPLEx extractions, allowing simultaneous collection of metabolites, proteins, and lipids, were used to prepare samples for metabolomic, proteomic, and lipidomic analysis [22]. Single and pooled (3 or 5 combined) SSAs printed with NAG or chitin as described above were extracted. A total of 6 samples were extracted via MPLEx. The standard extraction procedure was followed with the addition of two 45 s cycles of disruption with sterilized glass beads on an OMNI bead ruptor (OMNI International Inc., Kennesaw, GA, USA) to disrupt the SSA. Metabolite, lipid, and protein fractions were then prepped following standard procedures [22] and then stored at −20 °C until analysis. Fractions were analyzed for metabolite, protein, or lipid presence and to confirm no significant contamination from the gelatin methacrylate polymer. Metabolite fractions were analyzed in m/z range of 50 to 550 on an Agilent GC 7890A coupled with MSD 5975C mass spectrometer (Agilent Technologies, Santa Clara, CA). Protein concentrations were first measured using a Pierce BCA protein assay kit (Thermo Fisher Scientific, Waltham, MA, USA) and digested using a TFE (2,2,2-Trifluoroethanol) protocol [23]. Protein fractions were analyzed on a Q Exactive HF-X mass spectrometer (Thermo Scientific, San Jose, CA, USA). Lipid fractions were analyzed on a Waters NanoAquity UPLC system interfaced with a Velos-ETD Orbitrap mass spectrometer (Thermo Scientific, San Jose, CA, USA) in negative ionization. 

### 2.8. Biogeochemical Analyses in Synthetic Soil Aggregates

We first tested compatibility of SSAs with standard soil biogeochemical assays. Extracellular enzyme assays are a common soil biogeochemical assay that measure activity of extracellularly produced enzymes from soil microorganisms through the degradation of fluorescently labeled substrates [24]. These assays provide excess substrate availability for enzyme activity and are therefore measures of enzyme potential, not absolute activity. We applied this technique to SSAs to measure potential chitinase activity. Single SSAs containing *E. coli* RFP, *P. fluorescens*, or a co-culture of the two were printed as described above into individual wells of a black, 96-well plate. In total, SSAs were printed into 32 replicate wells and surrounded with 200 µL of MOPS minimal media. Sixteen replicate wells received 50 µL of 200 µM 4-methylumbelliferyl N-acetyl-β-D-glucosaminide (NAG-MUB), a fluorogenic substrate designed to report NAGase/chitinase activity upon enzymatic glycosidic bond hydrolysis and subsequent fluorescence. The remaining eight replicate wells received 50 µL of the fluorogenic standard, 4-methylumbelliferone (MUB), in standard concentrations to determine quench coefficients. Our assays also included eight replicates each of blanks (200 µL MOPS media with 50 µL DI water) and negative controls (200 µL MOPS media with 50 µL NAG-MUB substrate). Activity values are reported as nmol activity h^−1^ and calculated based on a 3-point MUB standard curve. Following NAG-MUB substrate addition, microplates were incubated at 23 °C for 18 h and then read on a Synergy H1 microplate reader (BioTek Instruments Inc., Winooski, VT) at an excitation of 350 nm and emission of 430 nm. 

We also examined the potential for SSAs to demonstrate biogeochemical processes via biosensors. The *E. coli* GFP used in this study was specifically engineered to produce urease for urea hydrolysis [18,19]. Urease activity of this organism changes its growth media’s pH from 6.8 to 8+ in the presence of urea. This allowed us to validate microbial activity using urea-containing growth media mixed with Bromothymol blue pH indicator which changes from yellow to blue in this pH range. 

### 2.9. Spatial Organization of a Synthetic Community within SSAs

In previous work by Lee et al. (2020), cheater–producer interactions between the chitin degrading strain *P. fluorescens* GFP and the non-chitin degrading strain *E. coli* RFP were studied. Spatial patterns in liquid media differed when these cultures were grown together in media containing chitin beads (heterogenous distribution) or homogenously distributed chitin monomers [17]. Drawing on this previous work, we explored the impact of a structured environment on the spatial organization of these co-cultured species and in the presence or absence of chitin.

After printing single, co-culture SSAs as described above into a 96-well plate, either 200 µL of MOPS + 50 µL DI water or 200 µL of MOPS + 50 µL NAG-MUB was added to each well to keep the SSAs hydrated and provide opportunities for competition or cheating between species. We used confocal fluorescent microscope images, taken after ~20 h of growth as described above, to compare the spatial distributions of bacterial cells/colonies in SSAs. Additionally, we used microscopy to the compare the spatial distributions of bacteria in SSAs to bacteria grown in liquid culture and to 2 µm fluorescent beads in SSAs. 

## 3. Results and Discussion

### 3.1. Characteristics of Synthetic Soil Aggregates

**Figure 1:**
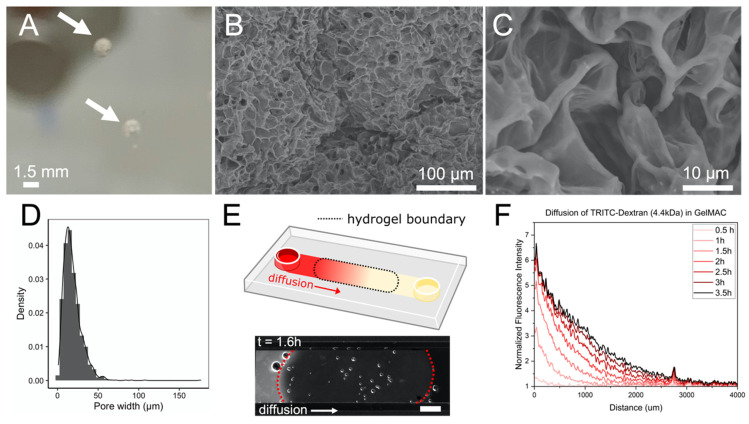
SSAs mimic the properties of natural soil aggregates. (**A**) A macroscopic perspective of the translucent SSAs (indicated by white arrows). (**B**,**C**) Scanning electron microscope images illustrate the heterogenous pore network within an individual SSA. (**D**) The pore size distribution within an SSA has a mean value of 18.5 µm and a standard deviation of 11.6 µm. (**E**) GelMA C was loaded into a fluidic channel (top) and the diffusion of 4.4 kDA TRITC-dextran through the hydrogel was tracked using fluorescence microscopy (bottom, hydrogel boundaries outlined in red, scale bar shows 2 mm). (**F**) The diffusion of 4.4 kDA TRITC-dextran through the GelMA C over time was used to calculate the diffusion coefficient of the compound through the hydrogel.

The SSAs were designed as spherical prints approximating 2 mm in diameter to replicate the overall size and shape of a natural soil macroaggregate (Figure 1). This diameter can be tuned and depends on the bioprinter resolution, the needle tip gauge, the printing pressure, and the hydrogel temperature and composition. The SSAs were printed using a translucent hydrogel (GelMA C, Cellink) to allow for optical imaging within single SSAs. Scanning electron microscope images show that this hydrogel resulted in a porous micro-structured environment inside each SSA. This heterogeneous pore structure produced an average pore size of 18.5 µm with a standard deviation of 11.6 µm. This pore size spread is comparable to the range of pore sizes that has been characterized within natural soil macroaggregates using X-ray microtomography (2–20 µm) [25]. 

Pore size is an important factor affecting soil microbial communities because it represents the available space for microbial growth. Macroaggregate pores can provide shelter to resident microbes to protect them from predators [26]. As pore size decreases, its complement, surface area, increases. High surface area within a soil macroaggregate provides increased opportunity for cellular attachment and activation of biofilm-relevant genes and phenotypes [27]. Pore size also has the potential to influence evolutionary dynamics within a microbial community. Smaller pores, such as the 18.5 µm average diameter pores measured in the SSAs, are likely to have smaller microbial populations than larger pores [28,29] and thus have a higher probability of genetic drift due to error in the small population’s sampling size. Similarly, a microbial community within a single pore could expect a stronger incidence of horizontal gene transfer due to spatial proximity [30]. In short, pore size offers the microbial community the opportunity to carve out a unique spatial and genetic niche.

However, individual pores are not completely isolated from the rest of the porous network within a soil aggregate. Hydraulic transport within a partially saturated soil aggregate is expected to temporarily connect individual “microbial villages” and allow the exchange of nutrients and biomolecules [11]. To measure the rates of molecular communication between microbial colonies in different pores, we tracked the diffusion of a small fluorescent molecule (4.4 kDa TRITC-Dextran) through the GelMA C. The diffusion coefficient of this molecule through GelMA C was calculated to be between $5.5\times 10^{-5}$ cm^2^ s^−1^ and $8.5\times 10^{-5}$ cm^2^ s^−1^. This diffusion coefficient is relatively fast and comparable to the diffusion in water of various bacterial exopolysaccharides, suggesting that, for lower molecular weight solutes, the diffusion of water through the hydrogel is the rate-limiting step for mass transport [31]. While diffusion rates have not yet been quantified within a natural soil aggregate, diffusion through bulk soil columns can be two orders of magnitude slower ($1.2\times 10^{-7}$ cm^2^ s^−1^ for Cl^−^), which may be attributed to other mass transport mechanisms such as molecular adsorption to surfaces [32]. The rapid diffusive transport within the SSAs provides an opportunity to use SSAs for the high-throughput study of dynamic interactions amongst microbial communities, such as metabolic complementation and bacteriocin warfare [33] and could be used to rapidly perfuse different media, antibiotics, or chemical fixation agents into the SSAs. 

The use of synthetic soil environments to mimic the microscale structure of the soil has been gaining interest in the field, most predominately as microfluidic devices (Aleklett et al. 2018; Stanley et al. 2016). These devices allow researchers to control all aspects of the soil environment, allowing systematic manipulations of microbial growth conditions, and have multi-modal analysis capabilities [34]. Though these systems represent an outstanding advancement for soil microbial ecology, they are not without limitations. For example, these systems can be time-intensive to produce, requiring specialized facilities and equipment to design photolithography masks and maintain clean environments. These systems are also limited to a 2-dimensional conformation, and thus microbial interactions occur only in one plane rather than in a realistic 3D environment. Microfluidics are also created using bio-inert polymers like polydimethylsiloxane (PDMS). Unlike synthetic soils created with microfluidics [34,35,36,37], bioprinted SSAs can be rapidly fabricated for high-throughput analyses and are created with a soft hydrogel-based polymer that can be consumed and restructured by resident microbes.

### 3.2. Microbial Viability within Synthetic Soil Aggregates

To test the compatibility of the bioprinting process with microbial viability and growth, we selected a diverse set of microorganisms to print into SSAs. Three Gram-negative bacteria, including two *Escherichia coli* (one modified to express urease) and a *Pseudomonas fluorescens*; one Gram-positive bacteria, *Paenibacillus* *polymyxa*; and one fungal species, *Neurospora crassa,* were successfully printed into the SSAs (Table 1). The optimal growth period, defined here as the length of time before which all microbial cells grow entirely within the SSA, differed depending on the organism and ranged from less than 24 h for *N. crassa* to up to 72 h for *P. fluorescens* and *P. polymyxa*. 

**Figure 2:**
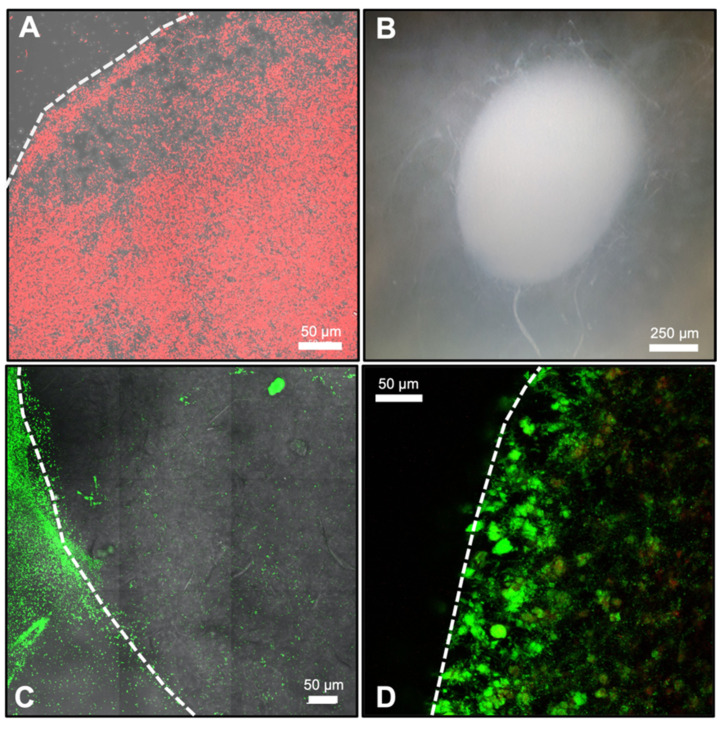
Microbial growth within SSAs. (**A**) Confocal fluorescent microscope image of *Escherichia coli* Δch1A within an SSA. Bacteria are tagged with red fluorescent protein and can be seen represented in red. The white dashed line shows the upper left boundary of the SSA structure. (**B**) Light microscope image of a SSA printed with *Neurospora crassa* spores. This image shows hyphae fully colonizing the SSA interior and expanding beyond the structure. (**C**) Confocal fluorescent microscope image of *Pseudomonas fluorescens* SBW25 at the SSA—media interface. Bacteria are tagged with green fluorescent protein and can be seen represented in green. The white dashed line represents the edge of the SSA, where bacteria to the right of this line are within the SSA. (**D**) Confocal fluorescent image of *Paenibacillus* *polymyxa* DSM 36 in an SSA. This strain was visualized using live/dead staining, where live bacteria are represented in green and dead bacteria are represented in red (an alternatively colored image is also available as Figure S2). The white dashed line represents the left boundary of the SSA. All SSAs were surrounded by liquid media during incubation and imaging.

Overall, we demonstrated that pure cultures of bacteria and fungi could be successfully printed into and grown in SSAs and observed via confocal fluorescence microscopy without additional sample preparation (Figure 2). However, fluorescence was essential for visibility due to the refractive index of the SSA hydrogel material. Fluorescence was successfully achieved either through the use of constitutive fluorescent protein producing microorganisms or by treatment with fluorescent dyes such as live/dead staining. This dual compatibility with fluorescent-protein-producing or fluorescently stained microorganisms increases the possibilities for visualizing a wide range of microorganisms within the SSAs, including species that are difficult to genetically modify. 

This range of microorganisms provides strong evidence that diverse and broadly representative soil microorganisms can be printed and studied within SSAs. Moreover, a range of ecological niches and physical behaviors can be explored, as with the saprophyte *N. crassa*. *N. crassa* is found globally, but frequently in subtropical regions, and is often associated with burned plants, a highly structured environment and nutrient source [38]. The powerful turgor required to move through plant structures may contribute to their rapid progression through the SSAs. Thus, there is high potential for native soil and environmental microbiomes to be printed and studied within these systems. SSAs may be uniquely valuable to the study of previously uncultured microorganisms. By providing an environment that mimics both the structure and chemical composition of soils, distinct microbial niches may be recreated within SSAs that otherwise cannot be replicated in the lab. This may increase our understanding of rare microbial taxa, which are thought to be the major drives of ecosystem function, but are difficult to study due to their low abundances [39]. 

### 3.3. In Situ Metaphenomics 

**Figure 3:**
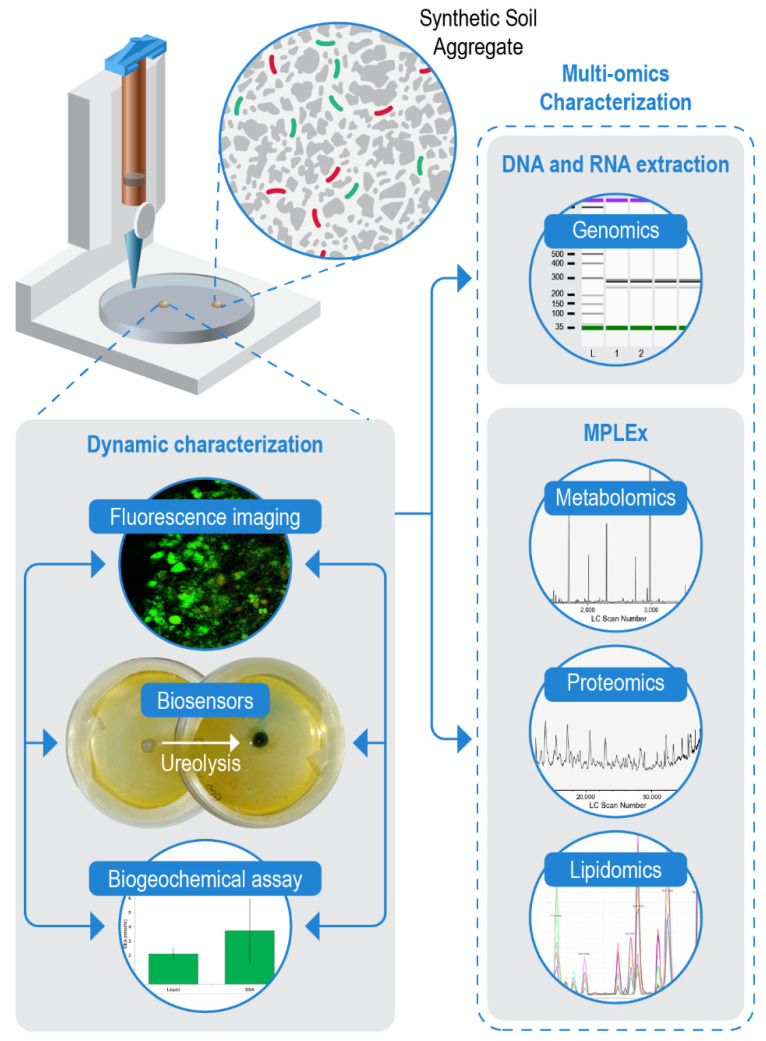
A schematic workflow for integrating SSAs with multi-modal analysis. All aspects of this workflow were validated in this study. SSAs with bacteria or fungal spores are printed into Petri dishes for growth. Dynamic characterization, including fluorescent images, biosensor application, and biogeochemical assays, can be performed once cells reach target growth. The presented fluorescent image is derived from Figure 2D. The biosensor image shows pH color change following ureolysis within a SSA (Figure S3). The biogeochemical assay shows example results from extracellular chitinase enzyme assays (see Figure S4 for full graph). All steps of dynamic characterization can be performed on the same SSA and in any sequence. Once dynamic characterization is complete, the same SSA can be then used for multi-omic characterization, including genomics, metabolomics, proteomics, and lipidomics. The genomics results show a gel electrophoresis image following 16S PCR amplification of DNA extracted from SSAs (see Figure S5). Metabolomics, proteomics, and lipidomics can be extracted simultaneously from a single SSA (see Figures S6–S8 for full total ion chromatograms).

Metaphenomics, or the study of a microbial community’s collective phenotype, can inform how environmental changes influence dynamic microbiome functions and processes [40]. This collective community property is measured using multi-omics including genomics, metabolomics, proteomics, transcriptomics, and lipidomics. In natural soil, interpreting these measurements can be challenging because non-relevant molecules (e.g., relic DNA and non-interacting chemical compounds) can obscure the active community metaphenomes [41]. Because the SSAs are fully chemically defined, we anticipated that we would be able to measure -omics properties from single and replicate pooled SSAs without the confounding background noise and variability of a natural soil environment. To this end, we optimized multi-omics protocols for single and replicate pooled SSAs using genomics, metabolomics, proteomics, and lipidomic analytical methods (Figure 3). 

For genomics analysis, we tested whether bacterial DNA could be extracted from SSAs, as this is the limiting step for genome and 16S sequencing. Bacterial DNA was successfully extracted from single and pooled SSAs using two commercially available kits. Total extracted DNA from single SSAs after 24 h ranged from 125 to 286 ng µL^−1^ but did not differ significantly by extraction kit used (F = 0.565, *p* = 0.531). Total extracted DNA from SSAs (single and pooled) after 48 h ranged from 279 to 625 ng µL^−1^ but did not differ significantly between single and pooled samples (F = 0.181, *p* = 0.172). Total extracted DNA tended to be greater for samples that were cultured for 48 h vs. 24 h, but this was not a significant difference (F = 6.680, *p* = 0.061). DNA was shown to be generally high-molecular weight and 16S genes were successfully amplified from all samples (Figure 3; Figure S5), indicating that this was reasonable quality DNA from bacteria and not contamination. 

The ability to extract microbial DNA from single, natural soil aggregates could provide invaluable sub-millimeter scale data, but achieving adequate yields from single soil aggregates is a challenge and often requires that aggregates be pooled despite their intrinsic variability [10,42]. Thus, truly microscale measurements of microbial structure and function remain elusive in natural soils. Our successful extraction of microbial DNA from single SSAs using standard kit protocols highlights the value of these systems to understanding fundamental aspects of soil microbial ecology. These SSAs provide opportunities for sub-millimeter analysis of microbial communities in structurally soil-like environments to address questions from microbial structure–function relationships to the evolution of microbial communities in soils. 

Next, we targeted metabolites, proteins, and lipids for analysis from SSAs. Because these compounds are frequently cross-referenced in the interpretation of microbial community properties, we used an established MPLEx preparation method to simultaneously extract metabolite, lipid, and protein layers from the same SSA sample (Figure 3). Following extraction, the protein layer was quantified using a BCA assay and was found to yield 42.2–51.5 µg of protein. As described in the methods, this extraction followed the standard MPLEx procedure with an added bead-beating step to homogenize the SSAs. We determined that this bead beating step was necessary to achieve adequate yields as we observed SSAs extracted without this additional homogenization yielded approximately 10x lower protein (2.6–4.6 µg). All metabolite, lipid, and protein samples were analyzed via mass spectrometry to confirm the presence of each of these ‘omics pools (Figure 3; Figures S6–S8). However, detailed annotation was not performed in this study because our emphasis was on protocol optimization for future work rather than re-characterization of well-defined model species. Future research should include the detailed characterization of microbial multi-omics extracted from SSAs.

These findings indicate that SSAs offer the potential to achieve single aggregate-scale multi-omic analysis of microbial communities. To date, this type of multi-omic extraction does not appear to have been applied at the single aggregate level for natural soils. Instead, larger quantities of soil (on the order of grams) are often extracted to generate these -omics pools [43,44,45,46]. Thus, SSAs have the potential to provide key sub-millimeter multi-omic insight. Such fine measurement scale data may allow us to quantify the link between microbial community composition and microbial activity, thereby improving microbial aspects of ecosystem models [10,47]. For example, SSAs could be employed as microbial bioreactors, allowing researchers to systematically apply treatments to individual aggregates to study microbial community responses [7]. 

While this initial proof-of-concept demonstrates the potential for multi-omics extraction from individual SSAs, we acknowledge that our limited replication does not definitively demonstrate the application of this method. Further study derived from this promising proof-of-concept and protocol optimization are needed to validate the use of this technique on SSAs. 

### 3.4. Biogeochemical Analyses and High-Throughput In Situ Assays

High-throughput biogeochemical assays are a common method of analysis for soil systems, including extracellular enzyme assays (EEAs), which measure the potential activity of hydrolytic enzymes excreted by plants and microorganisms into the soil [24,48]. These EEAs have been used to quantify extracellular enzyme activity of isolated soil aggregates and have shown that enzyme activity varies with aggregate size and aggregate isolation method [8,9,49]. To test the compatibility and ease of integration into existing methods of our SSAs, we performed a standard EEA protocol [24] to measure chitinase activity of individual or mixed bacterial communities in single SSAs printed into 96-well plates (Figure 3; Figure S4).

Compared to a liquid culture reference, we find chitinase activity to be measurable in printed SSAs, but only when *P. fluorescens* is in pure culture, suggesting that an interaction with *E. coli* RFP may reduce chitinase production by *P. fluorescens*. Previous observations of this cheater–producer pair suggest that in some cases the presence of *E. coli* RFP may negatively impact *P. fluorescens* growth when chitin is the primary resource [17]. Additionally, we noted that the variability in enzyme activity values was greater for SSA samples (CV = 18.2%) compared to liquid culture (CV = 4.5%). Variability in measured enzyme rates of soils is a known problem, even at the individual aggregate level [9,50]. Our findings suggest that spatial structure may play a role in this high variability and lend support to the realistic nature of SSAs. 

We also tested the potential for SSAs to function with biosensors. In this proof-of-concept, we used additions of a pH indictor dye to observe microbial ureolytic activity within SSAs. Single SSAs containing populations of *E. coli* GFP were printed into Petri dishes and surrounded by media containing urea and a bromothymol blue indicator. SSA color was observed to change from yellow-green (pH 6.8) to blue (pH 8) over a 24 h period, indicating urease activity by the printed bacteria (Figure 3; Figure S3). Because urea was only available in the surrounding media and not printed directly into the SSA, observed color changes of the SSA indicated diffusion of urea substrate through the SSA pore network. Additionally, we observed a small ring of color change in the media immediately surrounding the SSA suggesting potential for diffusion of ureolytic extracellular enzymes or byproducts out of the SSA and/or bacterial growth beyond the SSA. 

These soil aggregate proxies capable of incorporating biosensors have applications for the development of environmental monitoring and treatment assays. For example, in a soil environment that contains different microbes, the generation/consumption of metabolites can indicate the presence or absence of biological and chemical interactions. SSAs coupled with biosensing assays could thus inform in situ microbial distributions and interactions in edaphic environments. Whole-cell biosensors could also be applied to SSAs to detect heavy metal or pollutant contamination [51]. Thus, SSAs have the potential to provide rapid screening of sub-millimeter scale processes, including microbial activity. 

### 3.5. Synthetic Community Interactions in a Structured Environment

A heterogeneous structured environment is expected to a play a key role in shaping microbial metaphenomes through interactions such as spatial reciprocity, surface attachment, and biofilm production [35,52]. We explored the impact of 3D structure on the spatial interaction of two bacterial species (*P. fluorescens* and *E. coli*) shown to express a cheater–producer relationship when co-cultured in the presence of chitin oligomers [17]. 

**Figure 4:**
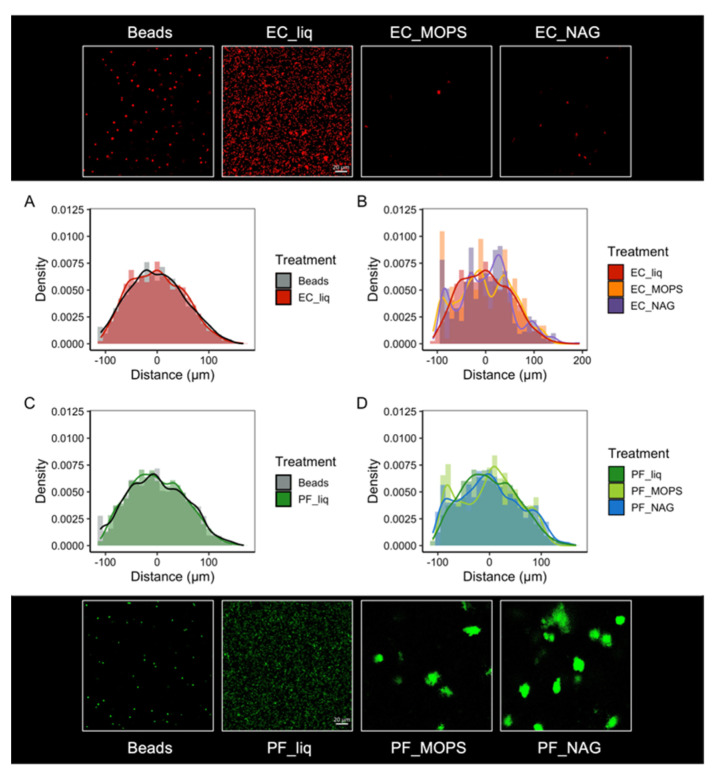
Spatial organization of *E. coli* RFP (EC) and *P. fluorescens* GFP (PF) cells within single SSAs compared to liquid culture and 2 µm fluorescent beads. (**A**,**C**) Histogram of bead–bead or cell–cell distances within SSAs vs. (**A**) EC and (**C**) PF in liquid culture. (**B**,**D**) Histogram of distances between (**B**) EC or (**D**) PF cells in liquid culture vs. in SSAs with MOPS-only media (MOPS) or MOPS with 4-Methylumbelliferyl N-acetyl-β-D-glucosaminide (NAG), a chitin proxy. Representative images are shown for each treatment. All images are 212 µm × 212 µm. Note that color saturation, brightness, and contrast of some images is enhanced for visibility in this figure (see Supplemental Material). EC and PF images and beads are shown as individual channels of the same SSA region.

We first showed that the spatial distribution of individual cells, relative to other cells of the same population, differs significantly when grown in the SSAs vs. in liquid culture (Figure 4; Table S1). As an abiotic control, we demonstrate that the spatial distribution of 2 µm fluorescent beads printed into SSAs differs significantly from the spatial structure of individual cells in SSAs, but not from individual cells in liquid culture (Figure 4). This finding indicates that the distribution of cells in SSAs is not due to an artifact of mixing the cultures with the hydrogel but is a direct result of microbial growth patterns likely driven by heterogenous resource distribution from the complex pore structures of the SSAs. 

**Figure 5:**
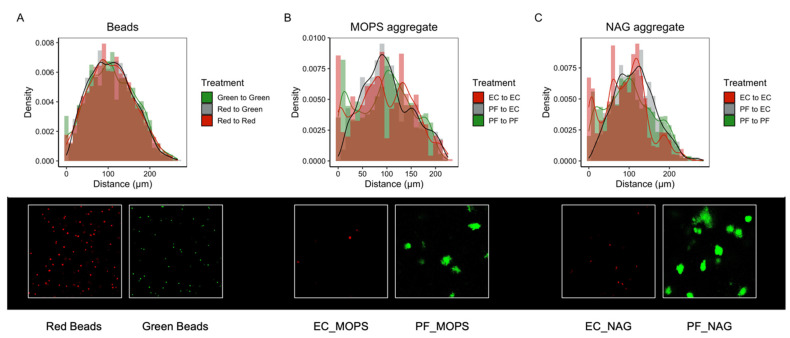
Interaction distances of *E. coli* RFP (EC) and *P. fluorescens* GFP (PF) cells within single SSAs compared to 2 µm fluorescent beads. (**A**) Histogram of bead–bead distances in an SSA. (**B**) Histogram of EC and PF interaction distances of cells printed in SSAs with MOPS-only media (MOPS). (**C**) Histogram of EC and PF interaction distances of cells printed in SSAs with MOPS containing 4-Methylumbelliferyl N-acetyl-β-D-glucosaminide (NAG), a chitin proxy. In all histograms, red represents EC to EC or red-to-red bead distances, green represents PF to PF or green-to-green bead distances, and gray represents EC to PF or green-to-red bead distances. Representative images are shown below each histogram. All images are 212 µm × 212 µm. Note that color saturation, brightness, and contrast of some images is enhanced for visibility in this figure (see Supplemental Material). EC and PF images and beads are shown as individual channels of the same SSA region.

**Figure 6:**
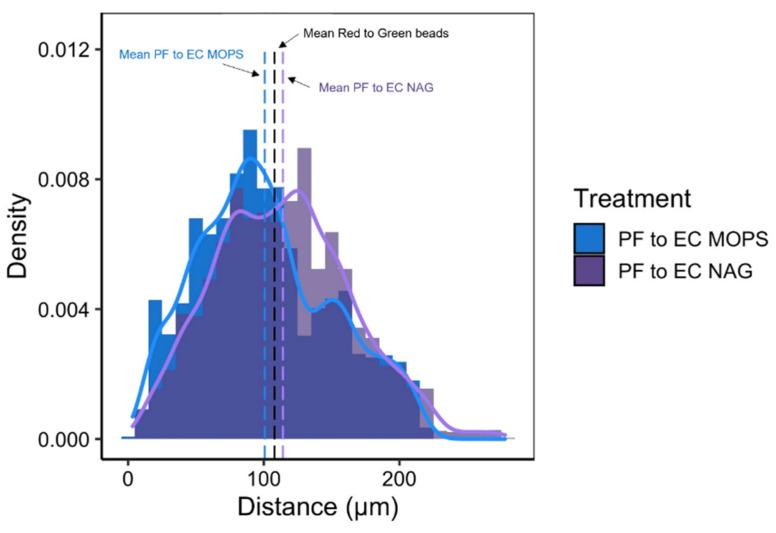
Histogram of *P. fluorescens* GFP (PF) to *E. coli* RFP (EC) cell distances within single SSAs surrounded by MOPS-only media (MOPS) or MOPS media containing 4-methylumbelliferyl N-acetyl-β-D-glucosaminide (NAG). Dashed lines represent the mean distance for MOPS (blue), NAG (purple), and fluorescent bead reference image (black).

Using the spatial distributions of each bacterial species (*E. coli* or *P. fluorescens*) relative to the other species, we explore co-culture interactions in a structured environment when in a predicted cheater–producer pair (MOPS minimal media with a chitin oligomer) vs. a predicted competitive interaction (MOPS minimal media). Our findings show that the spatial distributions between these species relative to each other differ significantly from spatial distributions of fluorescent beads and from the spatial distribution of cells relative to other members of the same population (Figure 5). We determined that the average distance between fluorescent beads was 107.9 µm, while the average distance between *P. fluorescens* and *E. coli* was less when grown in MOPS media at 100.7 µm and greater when grown in MOPS media with NAG at 114.1 µm (Figure 6). 

These results are evidence that a structured environment elicits unique microbial spatial organization, likely resulting from distinct microbial niches and gene x environment interactions, triggering emergent microbial metaphenomes. Thus, careful consideration of the micro-scale structure of the microbial environment is crucial to understanding and quantifying microbial metaphenomes. Our SSAs offer an alternative culturing method to liquid cultures that provides microbes an opportunity for niche establishment but remains multi-modal, allowing paired dynamic community characterization, such as imaging and biogeochemical assays, and multi-omic characterization (Figure 3). 

## 4. Conclusions

In this study, we developed and tested a novel application of 3D bioprinting technology to create SSAs, allowing rapid fabrication and experimental replication with multi-modal analysis compatibility. We first examined the potential of SSAs to mimic the physical structure of natural soil aggregates and ultimately found that the internal pore structure of SSAs was similar that observed for natural soil aggregates. Thus, SSAs can provide realistic microstructure for microbial growth. In turn, we found this microstructure influenced the spatial organization of two bacterial species, in pure and co-culture, compared to liquid culture. The SSAs are also highly tunable; although we did not parameterize hydrogel properties in this study, other studies have shown that changing the properties of the bio-ink can alter the internal pore network of the hydrogel, which may allow for further control over SSA permeability and pore structure [53]. We also anticipate that the SSAs can be amended with other solid phase compounds, such as plant litter or minerals, to simulate different nutrient inputs, mimic soil hotspots, and study microbe–mineral interactions. Integration of the SSAs into multi-omics workflows offers potential for correlating microbial ecology parameters such as community structure (e.g., genomics), properties (e.g., metabolomics, proteomics, lipidomics), and processes (e.g., chitin degradation rates). Further, single SSA resolution can be achieved with these multi-omic techniques, providing high-resolution correlation with similar resolution community dynamics data, such as fluorescence imaging and biogeochemical analyses. The strengths of SSA function and parameterization could help researchers forge an as-yet-unachieved high resolution understanding of the interplay between environmental properties and microbial ecology.

## Figures and Tables

**Table 1:** A list of bioprinted microorganisms and their growth period within the SSAs.

Microorganism	Validation Method	Optimal Growth Period
*Escherichia coli*	High-throughput kinetic read—Plate reader (OD600; RFP) Confocal microscopy—Red fluorescent protein	48 h
*Pseudomonas fluorescens*	Confocal microscopy—Green fluorescent protein	72 h
*Neurospora crassa*	Confocal microscopy—tdTomato fluorescent protein	<24 h
*Escherichia coli* (urea hydrolyzing)	Confocal microscopy—Green fluorescent protein Biosensor—Bromothymol blue pH indicator	24 h
*Paenibacillus* *polymyxa*	Confocal microscopy—Live/dead stain	72 h

## Data Availability

Data will be made available upon request.

## Supplementary Materials

The following supporting information can be downloaded at: https://www.mdpi.com/article/10.3390/microorganisms10050944/s1, Description of Image Adjustments, Figure S1: MorphoLibJ morphological segmentation image, Figure S2: Alternative color image of Figure 2D, Figure S3: Images of SSAs printed with ureolytic *E. coli* GFP, Figure S4: Extracellular enzyme activity of chitinase, Figure S5: Gel electrophoresis images, Figure S6: Total ion chromatograms from metabolomics, Figure S7: Stacked total ion chromatograms from negative-mode lipidomics, Figure S8: Total ion chromatograms from proteomics, Table S1: KS test results.
