# Supplementary material for: Synthetic Soil Aggregates: Bioprinted Habitats for High-Throughput Microbial Metaphenomics
Source: Microorganisms. 2022 Apr 30;10(5):944. doi: 10.3390/microorganisms10050944 (PMC9146112; doi:10.3390/microorganisms10050944)
Supplement: Supplementary file 1 [file microorganisms-10-00944-s001.zip › microorganisms-1685382-supplementary.pdf]

Supplemental Materials

Description of Image Adjustments: In figure 4 and figure 5 of the main text, the saturation, brightness, contrast of some confocal fluorescent images was adjusted to improve visibility. These include the EC_MOPS and EC_NAG image. For both images in both figure 4 and figure 5 adjustment were made as follows: Brightness: +40%, Contrast: +40%, Saturation: +400%.

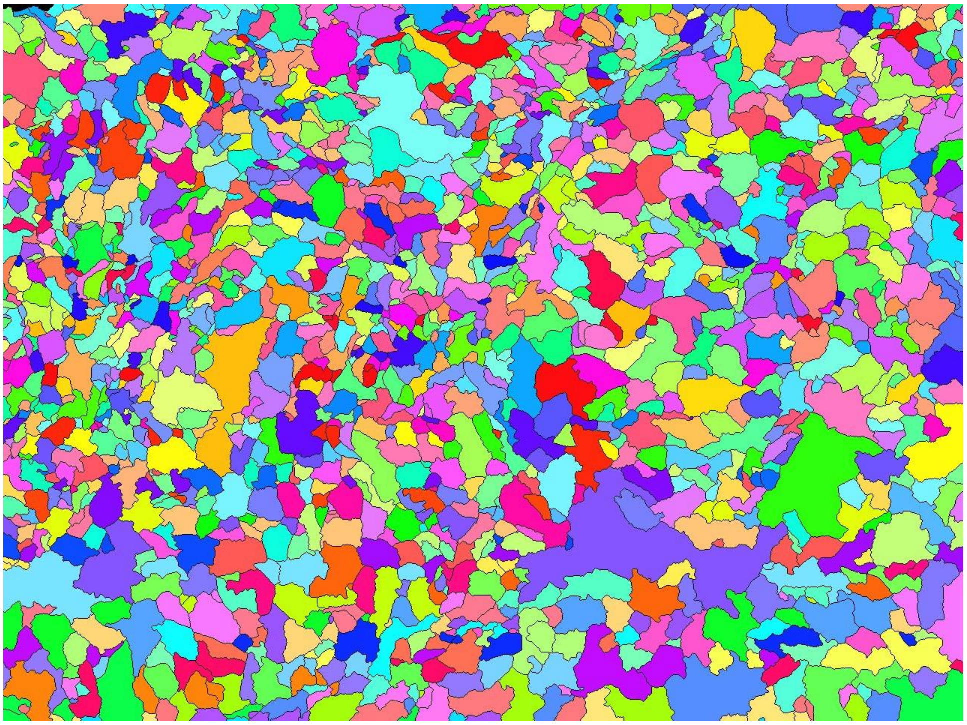

Figure S1: MorpholibJ morphological segmentation image.

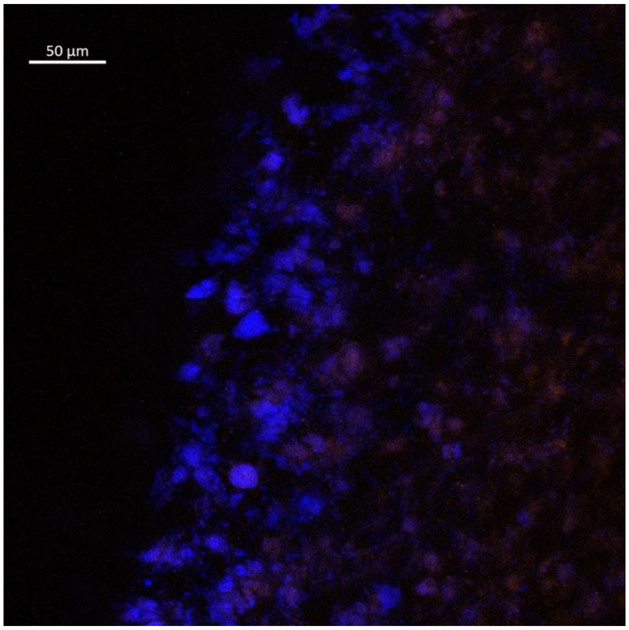

Figure S2: Alternative color image of Figure 2D.

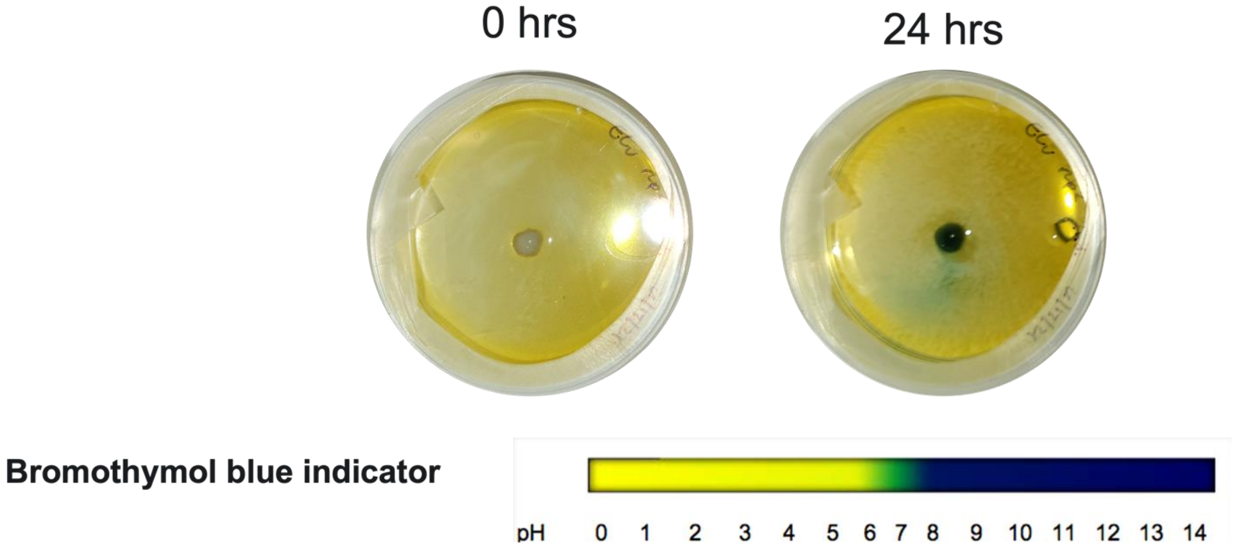

Figure S3: Images of SSAs printed with ureolytic *E. coli* GFP. Surrounding media contains urea, to support ureolysis, and bromothymol blue pH indicator. Images were taken 0 and 24 hours after printing. Color change is observed at 24 hours demonstrating a pH change indicative of ureolytic activity. Component of Figure 3.

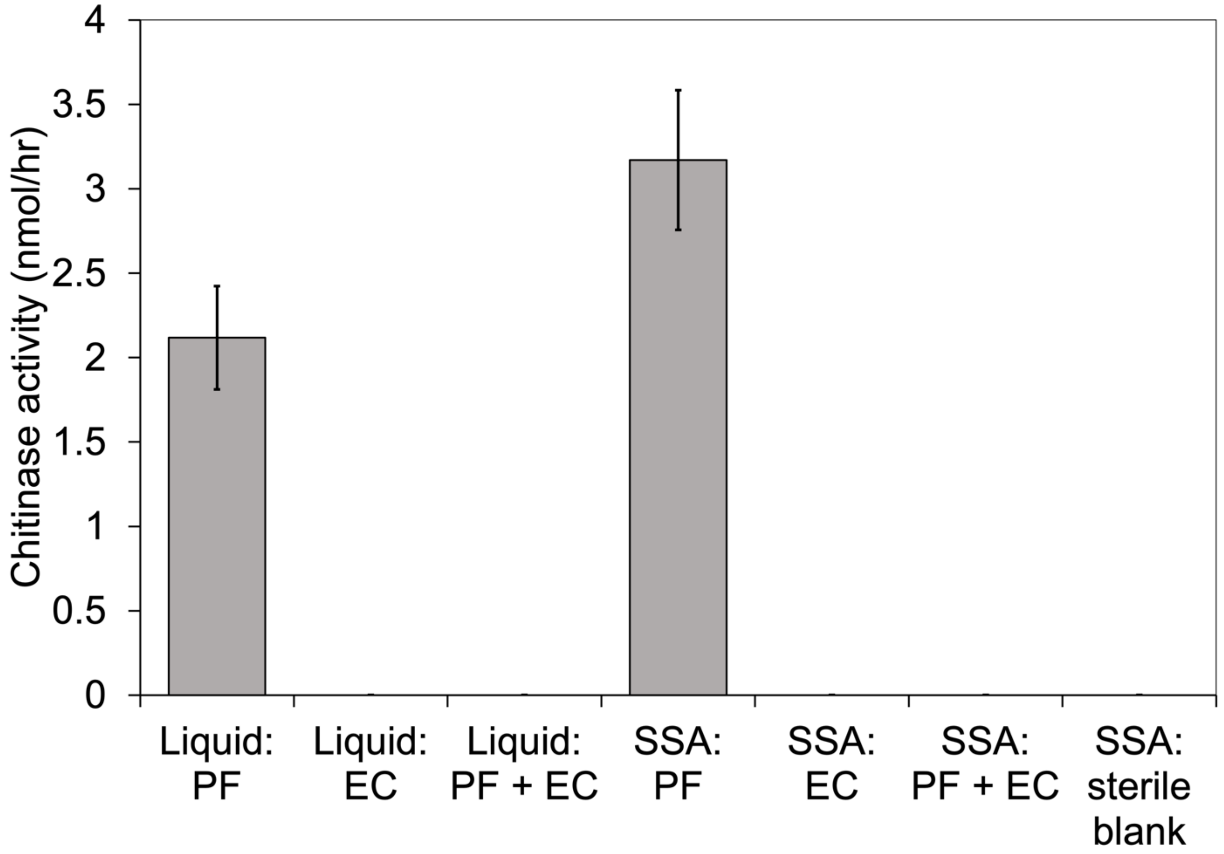

Figure S4: Extracellular enzyme activity of chitinase report in nmol activity hour^{-1}. Bars represent average activity $\pm$ standard error. Activity rates were only detectable in liquid culture of *Pseudomonas fluorescens* (PF) and in synthetic soil aggregates (SSAs) printing with PF. No chitinase activity was detected in the other treatments, including when *Escherichia coli* (EC) was present or when no bacteria were present (sterile blank). Component of Figure 3.

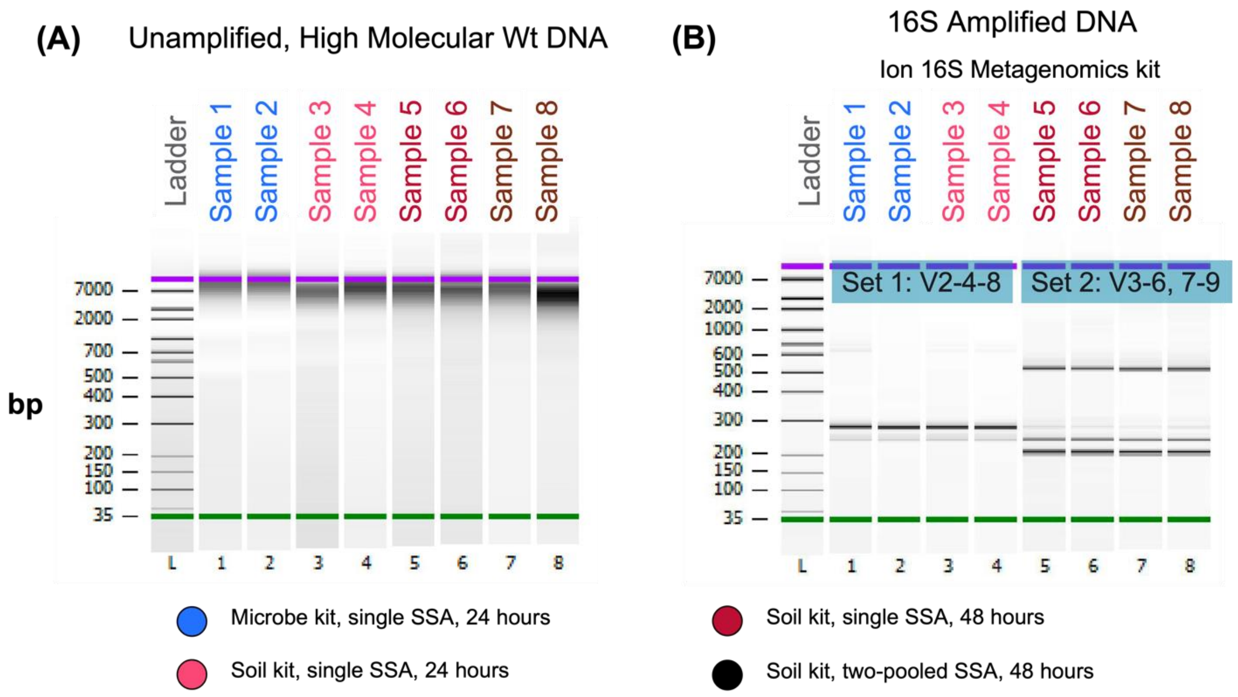

Figure S5: **Gel electrophoresis images** of (A) unamplified and (B) 16S amplified DNA extracted from single or pooled SSAs using commercial microbe or soil DNA extraction kits. Component of Figure 3.

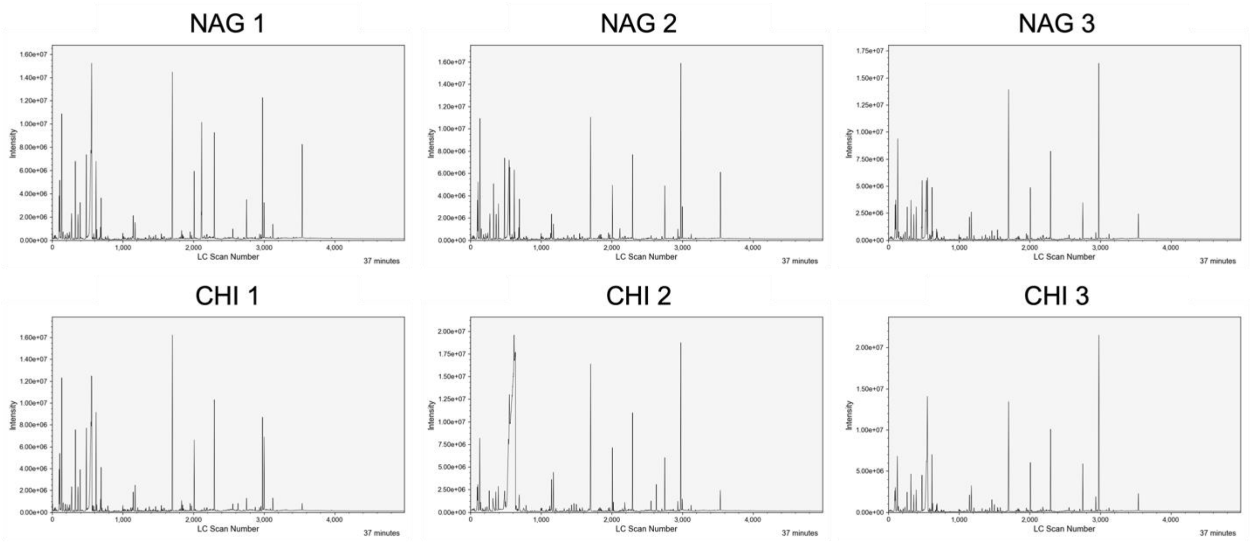

Figure S6: Total ion chromatograms from metabolomics analysis of SSAs with N-acetyl-β-D-glucosamine (NAG) or chitin (CHI) as a growth substrate. Component of Figure 3.

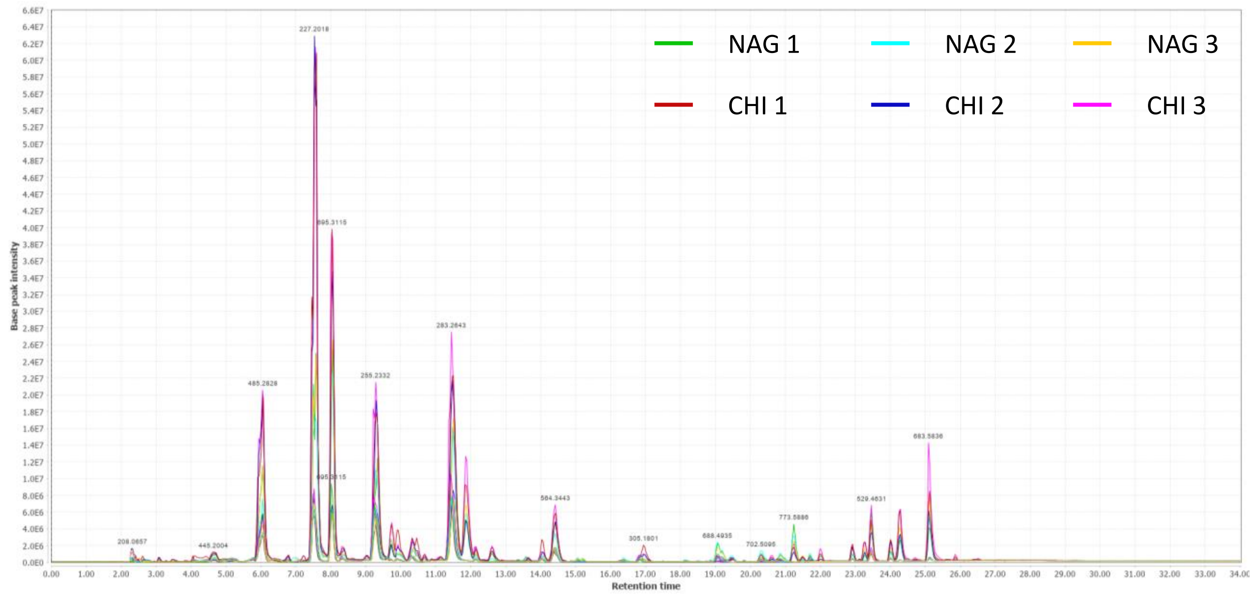

Figure S7: Stacked total ion chromatograms from negative mode lipidomics analysis of SSAs with N-acetyl-β-D-glucosamine (NAG) or chitin (CHI) as a growth substrate. Component of Figure 3.

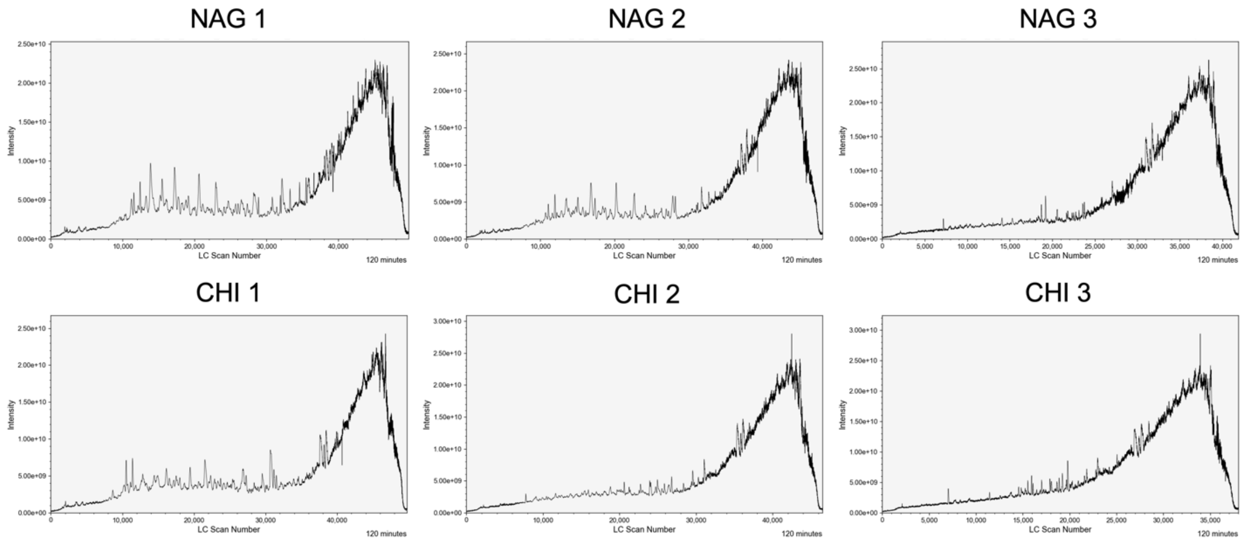

Figure S8: Total ion chromatograms from proteomics analysis of SSAs with N-acetyl-β-D-glucosamine (NAG) or chitin (CHI) as a growth substrate. Component of Figure 3.

Table S1. KS test results comparing spatial distribution of each group based on within population distances. NA indicates no direct comparison was made.

	FB green	FB red	PF liquid	PF NAG	PF MOPS	EC liquid	EC NAG
FB red	0.0576						
PF liquid	0.1848	NA					
PF NAG	**2.11 e -7**	NA	**1.46 e -8**				
PF MOPS	**< 2.2 e -16**	NA	**< 2.2 e -16**	**2.68 e -5**			
EC liquid	NA	0.5253	0.334	NA	NA		
EC NAG	NA	**< 2.2 e -16**	NA	**5.96 e -7**	NA	**< 2.2 e -16**	
EC MOPS	NA	**2.87 e -14**	NA	NA	**< 2.2 e -16**	0.2581	**1.52 e -14**
